# *BBX* Genes of *Cymbidium ensifolium* Exhibited Intense Response to Blue Light in Meristem Induction through Artificial Control

**DOI:** 10.3390/plants13172375

**Published:** 2024-08-26

**Authors:** Xiuming Chen, Muqi Niu, Xiaopei Wu, Yukun Peng, Ruiyue Zheng, Mengya Cheng, Kai Zhao, Yuzhen Zhou, Donghui Peng

**Affiliations:** 1 Cross-Strait Floriculture Industry Science and Technology Innovation Hub, Fujian Ornamental Plant Germplasm Resources Innovation & Engineering Application Research Center, Key Laboratory of National Forestry and Grassland Administration for Orchid Conservation and Utilization, College of Landscape Architecture and Art College, Fujian Agriculture and Forestry University, Fuzhou 350002, China; chenxiuming@fafu.edu.cn (X.C.); muqiniu@fafu.edu.cn (M.N.); wuxp7872@163.com (X.W.); pengyukun@fafu.edu.cn (Y.P.); ruiyuezheng@fafu.edu.cn (R.Z.); 18279880250@163.com (M.C.); 2 College of Life Sciences, Fujian Normal University, Fuzhou 350117, China; zhaokai@fjnu.edu.cn

**Keywords:** orchids, gene family, gene structure, blue light response, floral primordium induction

## Abstract

*Cymbidium ensifolium*, a prominent orchid species, is both highly valued for its ornamental qualities and commercially cultivated. However, the species has a considerable challenge in its breeding efforts due to the lengthy period of 7–8 years required for it to transition from seed germination to flowering. BBXs are multifunctional proteins that modulate the actions of critical regulators including *HY5* and *COP1* in response to blue light, ultimately impacting photomorphogenic processes. In this study, *BBX* proteins, known for their essential roles in regulating developmental processes under various light conditions, were chosen as the main subject of investigation. The outcome reveals the presence of 19 *BBX* genes in their genome. The genes are classified into four separate clades and dispersed among 12 out of the 20 chromosomes. Located in the nuclear, physicochemical properties of proteins, analysis of the promoter region reveals the existence of almost 800 cis-acting elements, highlighting the complex regulatory mechanisms that control the expression of the *CeBBX*s in various organs, as well as their response to light and hormone inputs. Moreover, the examination of differential expression under blue light therapy reveals their involvement in photomorphogenic reactions. The expression of *CeBBX*s exhibits substantial alterations as the duration of exposure to blue light increases. These findings contribute to a deeper understanding of the roles that *BBX* genes serve in *C. ensifolium*, providing a basis for future studies on the functions and regulatory mechanisms of *BBX* members in the context of floral initiation and development within this species.

## 1. Introduction

Unlike conventional artificial lighting systems, LED technology has evident strengths, such as a long lifespan, compact size, and higher photosynthetic efficiency. Consequently, LED technology has been extensively used in growing different agricultural crops under controlled conditions [1,2]. Recent studies have reported the use of different combinations of LED light spectra to regulate flowering times in horticultural crops, such as strawberries [3], *Chrysanthemum* spp. [4], *Petunia* spp. [5], and *Dahlia* spp. [6]. Research on strawberries has demonstrated that blue LED irradiation significantly promotes flowering [7,8]. Jeong et al. found that chrysanthemums treated with blue light as a supplementary light source formed flower buds 20.5 days after treatment. In contrast, flower buds could not be induced by white light treatment for 35 days [9]. These findings indicate that blue light-induced flowering in short-day plants can override the photoperiodic constraints on plant flowering regulation [8]. The flowering period is considered a critical agricultural characteristic because of the importance of floral features in ornamental horticulture. Furthermore, the utilization of optimized light treatments can enhance the process of plant hybridization and breeding, leading to an expedited flowering period, thereby improving breeding efficiency and the development of high-performing varieties. A comprehensive investigation has been carried out on the photoperiodic flowering pathway in plants. Accumulating data suggest that BBX proteins strongly influence the management of flowering time in Arabidopsis thaliana through photoperiodic mechanisms.

Zinc finger proteins (ZFPs) are abundant protein groups found in eukaryotes and have a vital function in the growth and development of plants [10]. A zinc finger is defined by the presence of cysteine and histidine residues. These residues coordinate around a zinc ion, resulting in the formation of a stable three-dimensional structure [11,12]. The BBX family is distinguished by the presence of the B-box domain. This domain, composed of around 40 amino acids, facilitates the creation of heterodimers with other members of the BBX family or different proteins, hence playing a crucial function in the control of transcription [13]. The B-box domain was initially identified in the *Xlxnf7* protein of the African clawed frog (*Xenopus laevis*) and has since been found in numerous BBX transcription factors in plants [14]. In contrast to animals, certain components from the BBX family in plants exhibit a remarkably preserved CCT (Constans, CO-like, and TOC1) as an area of focus [15], consisting of 42–43 amino acid residues, which has a role in controlling the transcription of BBX proteins and the movement of molecules between the nucleus and cytoplasm [16]. Furthermore, certain BBX proteins possess a Valine–Proline (VP) motif at their C-terminal end, which is involved in interactions with coiled-coil proteins [12,17].

*AtCO* was the first B-box protein to be cloned and identified. The modulation of *AtCO* gene expression and the stability of its protein are pivotal for the photoperiodic cues that trigger flowering, which, in turn, initiates the expression of the downstream *AtFT* gene [18,19]. *BBX4*, *BBX21*, *BBX22*, and *BBX23* are recognized for their ability to enhance photomorphogenesis [20,21,22,23,24]. Conversely, *BBX24*, *BBX25*, *BBX28*, *BBX30*, *BBX31*, and *BBX32* function as inhibitors of light signaling [22,25,26,27,28]. In *Cymbidium ensifolium*, *CRY2* can control cotyledon expansion and flowering time. *AtCRY2* has been shown to stimulate the expression of *AtFT* in response to blue light by inhibiting the degradation of the *AtCO* protein [29,30]. Under long-day photoperiods, mutants of the *AtCO* gene exhibit a significant delay in flowering time, whereas *AtCO* overexpression lines show an early flowering phenotype [31]. In *AtBBX7* overexpression lines, the transcription levels of *AtCO* and *AtFT* are inhibited under long-day conditions, resulting in delayed flowering, indicating that *AtBBX7*′s regulation of flowering time in depends on the *AtCO* gene [32]. Both *AtBBX4* and *AtBBX32* are negative regulators of flowering in *Arabidopsis thaliana*; the overexpression of *AtBBX4* and *AtBBX32* reduces *AtFT* expression levels and delays flowering under long-day photoperiods. *AtBBX4* loss-of-function mutants exhibit an accelerated flowering process under both long-day and short-day photoperiods, but the *AtBBX32* mutants do not demonstrate substantial variations in flowering time compared to the wild type under long-day circumstances [20,33]. Simultaneously, a growing body of research revealed the precise roles of BBX family members in different plant species, discovering BBX genes that concern the blooming time. Recently, researchers discovered nine individuals belonging to the BBX family that have a vital function in controlling the timing of blooming in rice [34]. Ye et al. identified *FaBBX29* as a significant contributor to the regulation of blooming [35]. Therefore, BBX proteins play a pivotal role in regulating the timing of flowering.

*Cymbidium ensifolium* is one of the most significant ornamental and commercial orchids in China, renowned for its graceful form, exquisite appearance, and fragrant aroma [36]. However, the growth cycle of this established orchid is long; it takes 7–8 years from seed to flowering under natural conditions, and it requires at least 10–12 years to breed new varieties with superior traits through cross-selection [37]. The extended breeding cycle of *C. ensifolium* significantly hampers the development and industrialization of new cultivars. While existing research indicates that blue light can be utilized to manipulate flowering time, and the BBX gene family is pivotal in modulating plants’ responses to blue light and their flowering time [35], its role in orchids remains unexplored. Consequently, identifying and elucidating the potential functions of BBX genes in orchids is of considerable importance. The objective of this study is to identify and characterize BBX family genes using genomic data from *C. ensifolium*. This includes analyzing their physicochemical properties, subcellular localization, gene structure, conserved domains, collinearity, evolutionary relationships, promoter cis-elements, and expression profiles. The results of this study provide new insights into the biological roles of BBX proteins in *C. ensifolium* and establish a foundation for future investigations into the connection between the blue light signaling pathway and BBX proteins. This could potentially shorten the breeding cycle, thereby enhancing the breeding efficiency and commercial viability of new orchid varieties.

## 2. Materials and Methods

### 2.1. Identification and Tertiary Structures of BBX Genes

With the aim of uncovering possible BBX genes in *C. ensifolium*, we acquired the BBX protein sequences of both *C. ensifolium* and rice from TAIR (https://www.arabidopsis.org/, accessed on 25 June 2024) [38] and RGAP (http://rice.plantbiology.msu.edu/, accessed on 25 June 2024) [39], respectively. The genomes of *C. ensifolium* were obtained by downloading the whole-genome sequencing data [40]. Initially, a BLASTp conduct was realized with the help of TBTools (http://cj-chen.github.io/tbtools, accessed on 25 June 2024), a software package that integrates multiple techniques and provides a handy approach for biologists [41]. The BBX domain (PF00643), which is conserved, was obtained according to the Pfam to conduct an HMMER survey with TBtools (http://pfam.xfam.org/, accessed on 25 June 2024) [41]. To further analyze the potential BBX proteins, the results from BLAST and Hmmsearch were subjected to examination (https://www.ncbi.nlm.nih.gov/Structure/bwrpsb/bwrpsb.cgi, accessed on 25 June 2024). Ultimately, proteins that possessed the entire BBX domain were selected and kept for further examination of their sequence (Table S1). The ExPASy proteomics server (https://www.expasy.org/, accessed on 25 June 2024)) was utilized to forecast the weight, quantization of protein unsteadiness, and hydropathicity index of the *CeBBX* proteins. Additionally, their position was forecasted [42,43]. In addition, to predict the tertiary protein structure of *CeBBX*, AlphaFold2.3.2 was utilized (https://colab.research.google.com/github/deepmind/alphafold/blob/main/notebooks/AlphaFold.ipynb, accessed on 25 June 2024). The SOMPA was employed when the width of output was configured to 70, and conformational status was established at 4 https://npsa-pbil.ibcp.fr/cgi-bin/npsa_automat.pl?page=npsa_sopma.html, accessed on 26 June 2024) [44].

### 2.2. Examination of the Exon–Intron Architecture and Identification

The conserved motifs of the *CeBBX* proteins were validated through the utilization of the MEME program (http://meme-suite.org/tools/meme, accessed on 25 June 2024) [45], while the conserved domain information was collected from NCBI. The exon–intron structure was examined using the genome-wide GFF annotation file, and visualization was performed using TBtools software [41].

### 2.3. Phylogenetic Analysis and Cis-Element Identification

The *BBX* proteins found in *C. ensifolium* and rice were utilized to categorize the *BBX* proteins specifically found in *C. ensifolium*. The Clustalx program was utilized for conducting diverse sequence correspondence of the entire set of BBX proteins [46]. The phylogenetic tree was constructed using the Neighbor-Joining (NJ) method with a thousand bootstrap replications, utilizing MEGA6.0 software [47]. Afterward, the tree underwent additional modifications using iTOL (https://itol.embl.de, accessed on 25 June 2024) [48]. The sequences located 2000 base pairs upstream of the transcriptional start sites and were designated as the proximal promoter region sequences. The promoter sequences of the *CeBBX* genes were obtained from the *C. ensifolium* Genomics Database. The promoter sequences were analyzed for cis-elements by submitting them to the PlantCARE database (http://bioinformatics.psb.ugent.be/webtools/plantcare/html/, accessed on 25 June 2024) [49].

### 2.4. Analyzing the Chromosome Position, Duplication Events, and Synteny of the CeBBX Gene in C. ensifolium

The genomes of *C. goeringii* were obtained by downloading its whole-genome sequencing data [50]. TBTools software was utilized to determine the positions of the *CeBBX* members on the chromosomes of *C. ensifolium*. Additionally, it was employed to visually represent the gene duplications of *CeBBX* genes and highlight regions that exhibit homology [41].

### 2.5. Plant Materials and Treatments

The plant materials utilized in this investigation were somaclones of *C. ensifolium* acquired from the Fujian Agriculture and Forestry University. Seedlings with three leaves were inoculated in a culture medium and subjected to different light treatments. The experimental plants were exposed to blue light, whereas the control group was subjected to white light. Leaf samples were collected at four specific time intervals: 0, 1, 3, 5, 7, and 15 days following the treatment. Three separate biological replicates were included in each sample collection to confirm the data’s trustworthiness. The specimens were swiftly frozen and preserved at a temperature of −80 °C for later extraction of RNA.

### 2.6. Extraction of RNA and Analysis Using Real-Time Quantitative PCR (RT-qPCR)

The FastPure^®^ Plant Total RNA Isolation Kit, designed for samples high in polysaccharides and polyphenolics, was sourced from Vazyme in Nanjing, China, for the purpose of extracting RNA. The concentration of RNA was measured with a Nanodrop 2000 spectrophotometer, and it was evaluated through agarose gel electrophoresis. For the conversion of RNA to complementary DNA (cDNA), the HiScript III 1st Strand cDNA Synthesis Kit, which includes a gDNA removal option, was also procured from Vazyme. The primers necessary for RT-*q*PCR were designed using the Primer3Plus online tool, with the actin gene serving as a control (Table S1). The RT-*q*PCR reactions were conducted with the Taq Pro Universal SYBR *q*PCR Master Mix Kit manufactured by Vazyme, located in Nanjing, China. The expression levels were determined using the 2^−∆∆Ct^ method [51].

### 2.7. Subcellular Localization of BBX Proteins

In order to ascertain the subcellular distribution of *CeBBX* in plant cells, the complete sequences of *CeBBX18* were integrated into the pCAMBIA1302-GFP vector. The recombinant vector was subsequently inserted into the *Agrobacterium tumefaciens* strain GV3101 (ANGYUBio, Fuzhou, China). Undamaged tobacco leaves (*Nicotiana benthamiana*) were chosen and infused with *A. tumefaciens* carrying the modified vector. The tobacco plants were placed in a dark environment and kept at a temperature of 25 °C for a duration of 12 h. Fluorescence images of GFP were acquired using a confocal scanning microscope called Axio-Imager_LSM-800, manufactured by Zeiss in Oberkochen, Germany.

## 3. Results

### 3.1. Identification and Characterization of BBX Proteins in C. ensifolium

**Figure 1:**
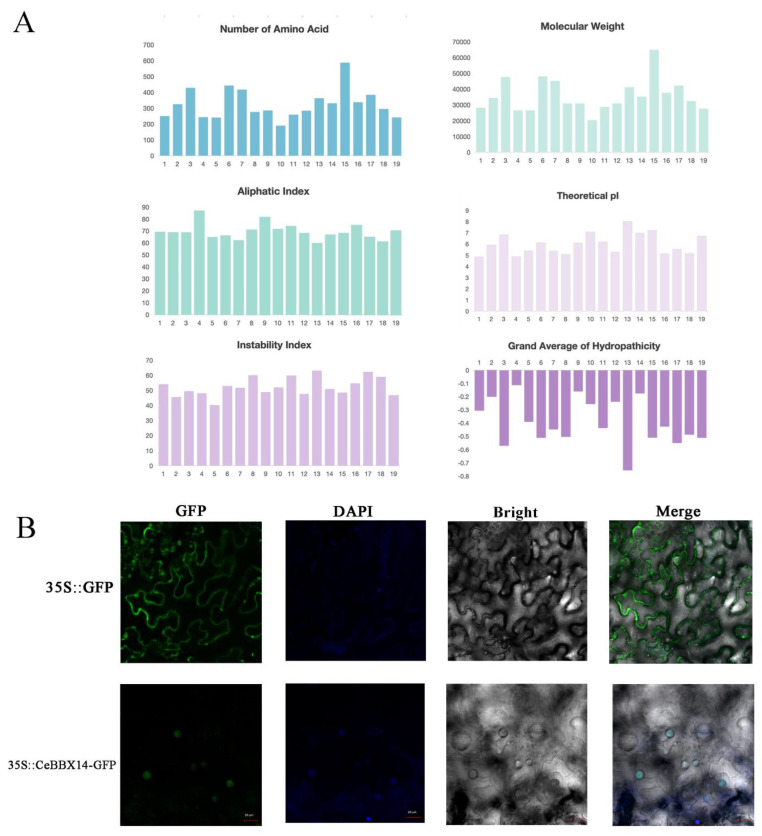
Identification and characterization of BBX proteins in *C. ensifolium.* (**A**) Physicochemical properties of 19 *CeBBX* proteins; (**B**) Subcellular localization of *CeBBX14*. The images, arranged from left to right, depict the green fluorescent protein (GFP), the DAPI field (representing nuclear staining), the bright field, and an overlay of GFP, DAPI, and brilliant field from the same sample. Scale bar = 20 μm.

A BLASTP search was conducted on the *C. ensifolium* genome database using *BBX* protein sequences from the same species to identify *BBX* family members. After eliminating duplicate sequences, 19 *BBX* genes were identified and designated as *CeBBX1* to *CeBBX19* based on their gene IDs in ascending order (Figure 1 and Table S1). The result shows detailed information for each *CeBBX*. The characteristics assessed included the count of amino acids, theoretical isoelectric point, and the average of GRAVY. The *CeBBX* proteins exhibit significant variation in size, with amino acid counts ranging from 189 (*CeBBX10*) to 587 (*CeBBX15*). Correspondingly, their molecular weights range from 20,281.83 Da (*CeBBX10*) to 64,770.75 Da (*CeBBX15*). The theoretical pI values span from 4.87 (*CeBBX1*) to 8.01 (*CeBBX13*), with most *CeBBX* proteins having a pI below 7, indicating they are predominantly acidic. Proteins with higher pI values, such as *CeBBX10* and *CeBBX13*, are more basic. The instability index varies from 40.06 (*CeBBX5*) to 73.86 (*CeBBX11*), suggesting that many *CeBBX* proteins may be prone to rapid degradation or require stabilization through interactions with other cellular components. The aliphatic index ranges from 59.42 (*CeBBX13*) to 86.79 (*CeBBX4*), with higher values suggesting greater thermostability. The GRAVY values range from −0.755 (*CeBBX13*) to −0.11 (*CeBBX4*), emphasizing that the proteins are generally hydrophilic. Subcellular localization analysis reveals that all *CeBBX* members are localized in the nucleus. Additionally, transient expression of a recombinant *CeBBX*-*GFP* protein in tobacco epidermal cells shows that *CeBBX18* is confined to the nucleus, suggesting that *CeBBX* proteins are involved in the regulation of nuclear transcription, consistent with their predicted subcellular localization.

### 3.2. Gene Structures, Conserved Domains, and Motif Analysis

**Figure 2:**
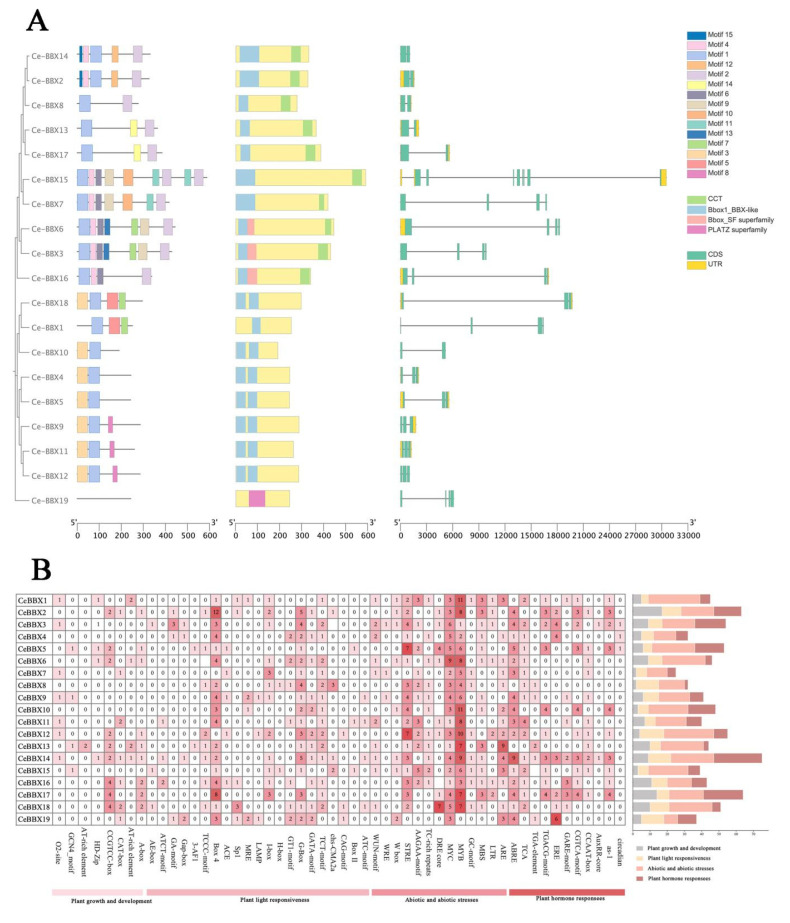
(**A**) Gene structures, conserved domains, and motif analysis; (**B**) Cis-element analysis of *CeBBX*s.

Our findings display the conserved patterns found in 19 *CeBBX* proteins (Figure 2A). BBX genes with comparable gene architectures and identical conserved motifs often group in the evolutionary tree. Among these 19 BBX genes, 15 conserved motifs have been identified. Notably, *CeBBX6* and *CeBBX3* each contain the highest number of conserved motifs, with seven. Motifs 1, 2, and 4 are notably recurrent across various *CeBBX* proteins, suggesting their pivotal roles in the proteins’ functions. *CeBBX15*, in particular, displays a diverse array of motifs, including Motif 5 and Motif 10, potentially endowing this protein with unique functional properties. Additionally, a total of 10 BBX genes possess a CCT domain. Comprehensive gene structure analysis reveals that all 19 BBX genes contain between one and four introns. Of these, *CeBBX18* has the longest intron, followed by *CeBBX6* and *CeBBX16*. The gene *CeBBX15* is marked by a complex arrangement of multiple exons. Conversely, *CeBBX14* has a simplified gene structure with fewer introns. *CeBBX6* and *CeBBX7* feature extended untranslated regions (UTRs), which might influence their mRNA stability and translation efficiency.

### 3.3. Cis-Element Analysis of CeBBXs

A thorough investigation of the promoter regions of the *CeBBX* gene family in plants was undertaken using the PlantCARE database (Figure 2B) to better understand their regulatory complexity and environmental responsiveness. A total of 800 cis-acting elements were discovered in the promoters of these genes, highlighting the intricate control of gene expression tailored to diverse environmental cues and internal physiological states. The distribution of these elements includes 230 light response elements, showcasing the significant influence of light on the gene expression of this family. Hormonal regulation is also profoundly represented by 202 hormone response elements, underscoring the sensitivity of these genes to hormonal signals. These include a range of specific elements, such as abscisic acid response elements (ABREs), gibberellin response elements (GARE-motifs), and auxin-responsive elements (AuxRR-cores), among others. Notably, salicylic acid response elements and jasmonic acid-responsive elements (MeJA-responsive elements), like the CGTCA motif and TGACG motif, were prevalent. This indicates a strong correlation between CeBBX gene expression and pathways involved in stress and defense mechanisms. In addition, 373 elements related to both abiotic and biotic challenges demonstrate the involvement of *CeBBX* genes in enhancing plant resistance to environmental pressures. Another 69 elements related to general growth and developmental processes emphasize the involvement of *CeBBX* genes in various aspects of plant development. Particularly, *CeBBX2*, *CeBBX14*, and *CeBBX19* had a high concentration of elements related to both light and hormones, suggesting their pivotal roles in light responses mediated by these hormones. *CeBBX14*, enriched with both light-responsive elements and ABREs, may indicate a dual regulatory mechanism influenced by light and abscisic acid, possibly coordinating photosynthesis with water stress responses. This detailed mapping and categorization of cis-acting elements reveal not only the potential functional roles of each *CeBBX* gene in response to environmental and developmental signals but also suggest a complex network of gene regulation that allows plants to adapt to their ever-changing environment.

### 3.4. Phylogenetic Analysis and Tertiary Structure Analysis of CeBBXs

**Figure 3:**
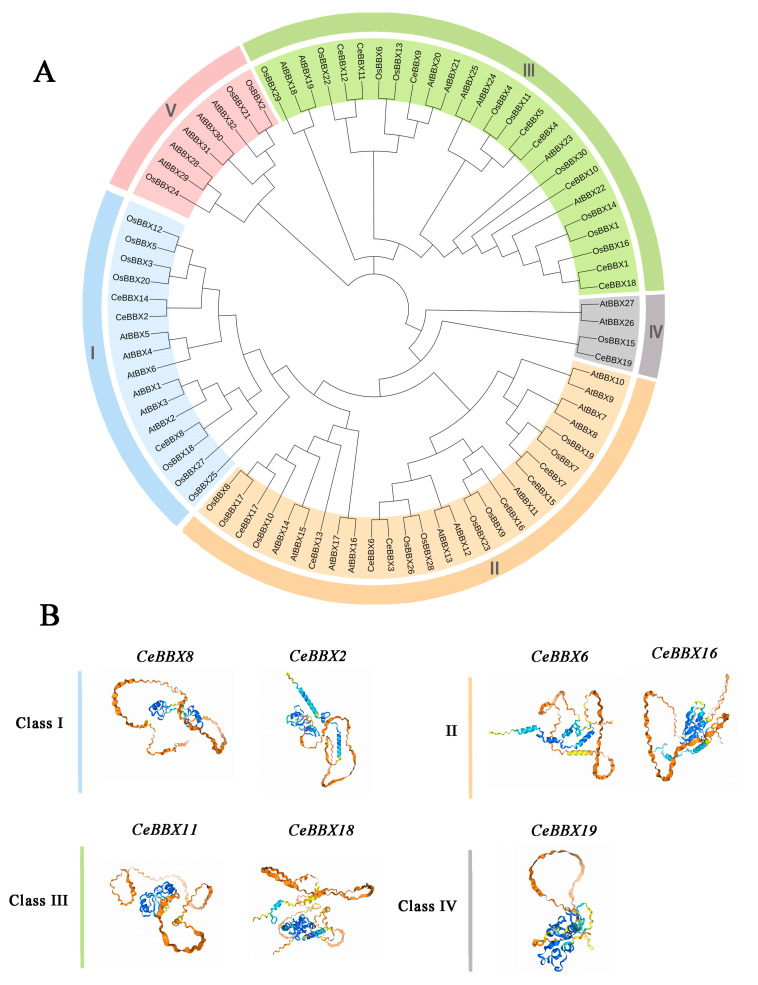
(**A**) Phylogenetic trees of *BBX* gene family in *Cymbidium ensifolium*, *Arabidopsis thaliana*, and *Oryza sativa*; (**B**) Anticipation of the three-dimensional structure for *CeBBX* proteins was analyzed. The proteins were categorized into Class I, II, III, and IV. The confidence levels of the protein structures are represented by lines of varying colors and red circles, with the confidence increasing from blue to orange. (Blue: pIDDT > 90; light blue: 90 > pIDDT > 70; yellow: 70 > pIDDT > 50; orange: pIDDT < 50).

Phylogenetic trees were created using the neighbor-joining method to investigate the evolutionary relationships and classification of the BBX gene family. The analysis included species such as *C. ensifolium*, *A. thaliana*, and *O. sativa*. (Figure 3A). The analysis identified 19 *CeBBX* proteins, 32 *AtBBX* proteins, and 30 *OsBBX* proteins, which were categorized into five distinct groups (I–V). Among these, Group II contained the highest number of members, including seven *CeBBX* proteins. This was followed by Group III with 26 members, Group I with 16 members, Group V with 8 members, and Group IV with 4 members. The congruence between the phylogenetic tree and the clustering results of prior investigations affirms the precision and dependability of the phylogenetic tree [52]. Interestingly, no *CeBBX* members of *C. ensifolium* were found in subfamily IV. Meanwhile, BBX proteins from *C. ensifolium*, *Arabidopsis*, and rice subfamilies were found to be closely related, indicating a high level of evolutionary conservation within the BBX family and suggesting that they may have comparable biological activities. The phylogenetic tree showed that the BBX protein of *C. ensifolium* was more closely related to its homology in rice than to its homology in *Arabidopsis*. The subsequent analysis provides a detailed prediction and examination of the structural prediction of secondary proteins (Table S2) and the tertiary structure of each branch of BBX proteins in *C. ensifolium* (Figure 3B). The proteins from different branches displayed notable variations in the amounts of alpha helices, extended strands, beta twists, and random coils. Additionally, proteins that clustered together in the evolutionary tree showed similar tertiary structures. The analysis reveals that the numbers of *α*-helices and *β*-strands in *CeBBX6* and *CeBBX16* are greater than those observed in proteins from other classes. *CeBBX18* is characterized by a notable number of α-helices and a minimal presence of visible *β*-strands, suggesting a helix-dominated configuration. Furthermore, the variations in length between *CeBBX11* and other Class III proteins could perhaps explain the disparities in their tertiary structures.

### 3.5. Chromosomal Location and Gene Duplication of CeBBX Gene

**Figure 4:**
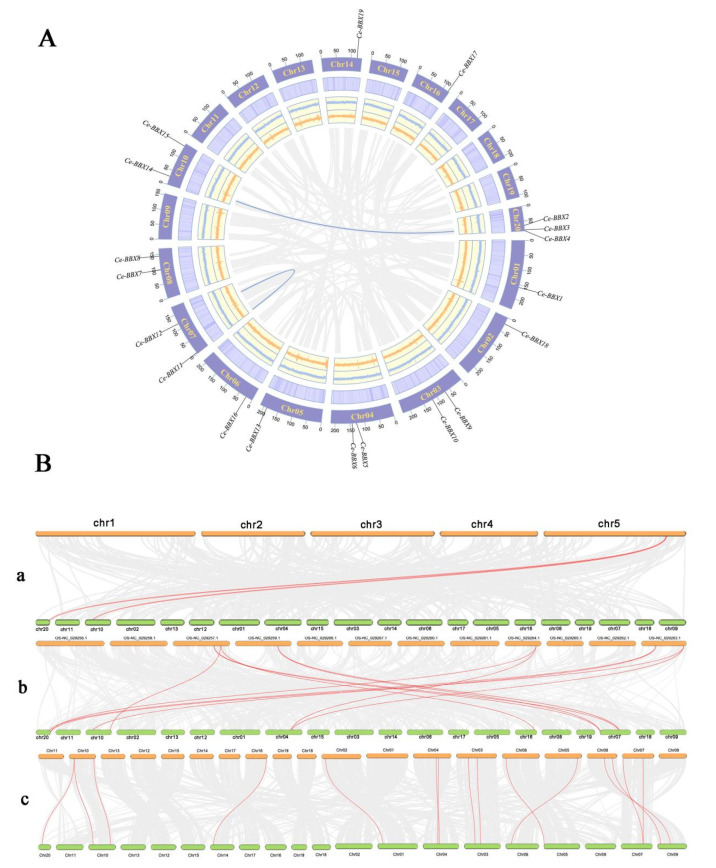
(**A**) Chromosomal location and gene duplication of *CeBBX* gene; (**B**) Chromosomal localization of 19 *CeBBX* genes. Collinearity analysis of *Cymbidium ensifolium* with *Arabidopsis thaliana* (**a**), *Oryza sativa* (**b**), and *Cymbidium goeringii* (**c**).

An investigation was conducted to determine the specific locations of the 19 *CeBBX* genes on the chromosomes of the *C. ensifolium* genome in order to understand how the *BBX* genes are distributed within the genome (Figure 4A). The 19 *CeBBX* genes were distributed across 12 out of the 20 *C. ensifolium* chromosomes, accounting for approximately 60% of the total chromosomes. Notably, chromosome Chr20 harbored the highest number of *CeBBX* genes, with a total of three. This was followed by chromosomes Chr03, Chr04, Chr07, Chr08, and Chr10, each of which contained two *CeBBX* genes. The remaining six chromosomes each housed a single *CeBBX* gene. The overall distribution of *CeBBX* genes across the *C. ensifolium* chromosomes was relatively even despite some variability in gene density.

Gene duplication events are essential for the proliferation of gene families and are a prevalent feature of plant evolution [37]. In the *C. ensifolium* genome, nineteen *CeBBX* genes were identified as participating in two segmental duplication events, specifically *CeBBX2*/*CeBBX14* and *CeBBX11*/*CeBBX12* (Figure 4A). All instances of duplicated genes were classified together in the same clade of the evolutionary tree, highlighting gene duplication as the main mechanism responsible for the growth of the *CeBBX* gene family. In order to obtain a more profound comprehension of the phylogenetic and evolutionary patterns of the *CeBBX* gene family, a comparative synteny study was performed on *C. ensifolium* and three other species: *A*. thaliana, *O. sativa* (rice), and *Cymbidium goeringii* (Figure 4B). The analysis reveals homologous gene pair counts of 2, 11, and 15 with *C. ensifolium*, rice, and *C. goeringii*, respectively. Notably, the monocotyledonous plant *C. ensifolium* exhibited a greater number of homologous gene pairs with rice and *C. goeringii* compared to the dicotyledonous plant *A. thaliana*, indicating closer phylogenetic relationships within the monocots. Furthermore, several *CeBBX* genes demonstrated homology with multiple genes in the aforementioned species, suggesting that these genes may play a crucial role in the diversification and functional expansion of the *BBX* gene family. This comparative genomic approach highlights significant evolutionary relationships and mechanisms that might contribute to the adaptive diversity observed in plant species.

### 3.6. Expression Characteristics of CeBBX in C. ensifolium under Different Lights

**Figure 5:**
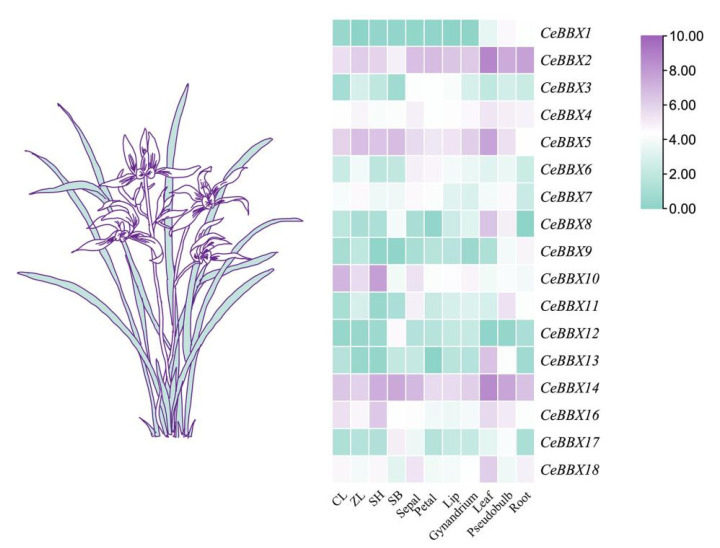
Heatmap of the expression patterns of the *BBX* gene family in wild *C. ensifolium* with different organs and different periods. Note: CL: initial bud period (1–5 mm); ZL: middle bud period (6–10 mm); SH: Full flowering period; SB: Flower decay period.

In the initial phase of this research, transcriptome data were collected for various organs of *C. ensifolium*. These included several vegetative parts, such as leaves, roots, and pseudobulbs, along with different segments of the reproductive structures, including sepals, petals, labellum, and gynandriums. These data enabled us to perform a comprehensive analysis of the transcriptome to assess the expression levels of the *CeBBX* genes. The investigations focused on the expression patterns of 18 *BBX* genes in *C. ensifolium* (Figure 5). Among these, *CeBBX9* and *CeBBX12* were characterized by low expression levels across all samples, with numerous instances of non-expression. Conversely, *CeBBX2* and *CeBBX14* showed significantly elevated expression levels relative to their counterparts. The expression of *CeBBX* genes exhibited notable tissue-specific patterns, with certain genes presenting prominent expression in specific tissues. Specifically, *CeBBX10* was highly expressed solely in flowers, while *CeBBX8* and *CeBBX13* were predominantly expressed in leaves. In contrast, *CeBBX11* was uniquely expressed in pseudobulbs. Additionally, the expression dynamics of the *BBX* gene family revealed a distinct pattern throughout the flowering phase of *C. ensifolium*. The expression levels of *CeBBX2*, *CeBBX3*, *CeBBX10*, *CeBBX16*, and *CeBBX18* showed a gradual decline as the flowers aged, whereas the expression levels of *CeBBX12* and *CeBBX17* reached their peak during the decay phase.

### 3.7. Expression Profiles of Cymbidium ensifolium BBX Genes with Blue Light Treatment

**Figure 6:**
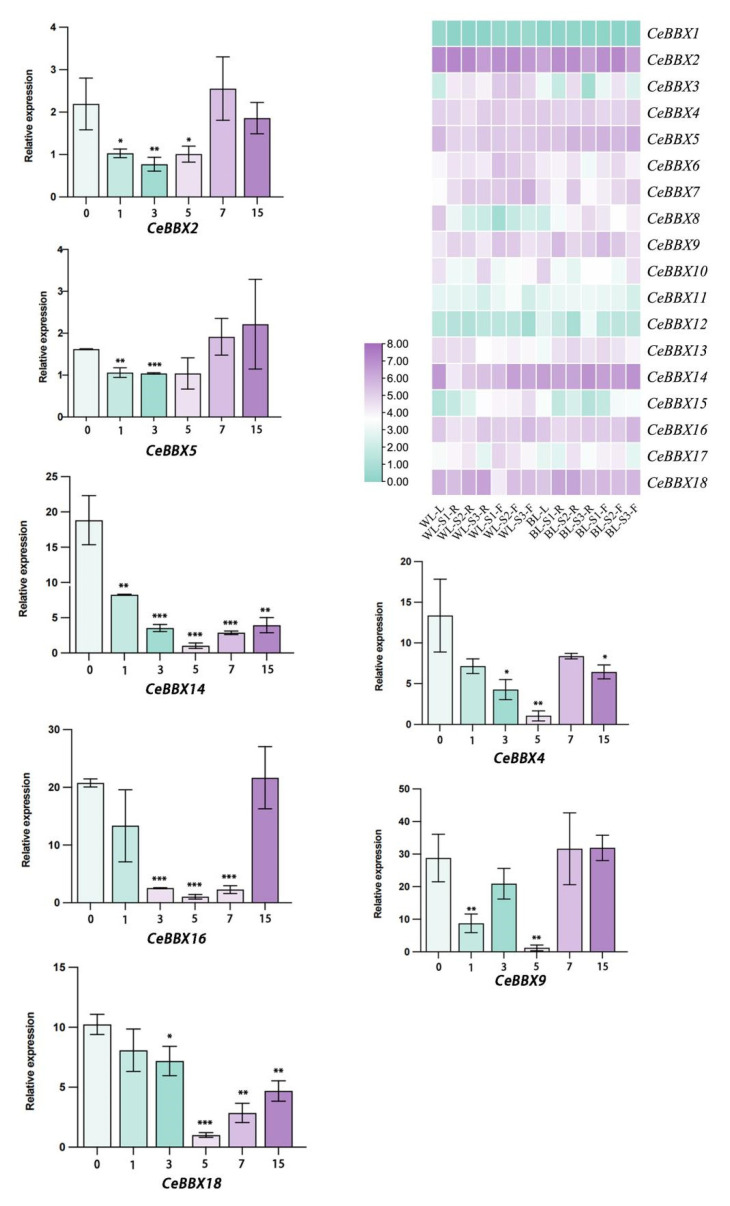
Seven *CeBBX* genes were analyzed using RT-*q*PCR. The transcriptome data of these genes were collected throughout four distinct growth stages of *C. ensifolium* under both white and blue light treatments. WL: white light, BL: blue light, F: flower, R: rhizome, L: leaf, S1: the flower bud phase, S2: the complete flowering time, and S3: the period when the flowers wither. The color scale indicates the logarithm base 2 of the normalized counts per million kilobases of reading. The data shown are the means ± standard error (SE) of three independent measurements. The statistical significance levels are represented as follows: a *p*-value less than 0.05 is designated by *, a *p*-value less than 0.01 is denoted by **, and a *p*-value less than 0.001 is denoted by ***.

Seven *CeBBX* genes (*CeBBX2*, *CeBBX4*, *CeBBX5*, *CeBBX9*, *CeBBX14*, *CeBBX16*, and *CeBBX18*) were selected based on their positions in the phylogenetic tree and their relatively high expression levels in leaves. Leaf samples were obtained at six different time points, 0, 1, 3, 5, 7, and 15 days following exposure to blue light. The relative expression levels of these seven *CeBBX* genes before and after treatment at different time points were analyzed using RT-*qPCR* (Figure 6). The results indicate that the expression levels of *CeBBX4*, *CeBBX14*, *CeBBX16*, and *CeBBX18* significantly decreased within the first five days, with all genes exhibiting lower expression levels compared to the control group. However, expression levels increased with prolonged blue light exposure. On the 15th day, the expression levels of *CeBBX2* and *CeBBX18* remained lower than those of the control group, while the expression levels of *CeBBX9* and *CeBBX16* were approximately the same as those of the control group, consistent with the transcriptome data. Furthermore, the expression level of *CeBBX5* was elevated compared to the control group on the 15th day, while the expression level of *CeBBX14* was notably reduced. Given that blue light influences plant morphogenesis and photoperiod, it is plausible that these changes in *BBX* gene expression are a direct response to blue light signaling, thereby regulating related biological processes.

## 4. Discussions

The *BBX* transcription factor family, ubiquitously present in plants, plays a vital part in numerous physiological processes, such as the control of blooming time, light signal transduction, and stress responses [53]. Over the past decade, advancements in genomic data and bioinformatics have facilitated the identification of *BBX* transcription factors across numerous species, with notable interspecies variation in the number of *BBX* family members [54,55]. In this study, we conducted a comprehensive analysis of the BBX gene family in the genome of *C. ensifolium*, identifying a total of 19 *BBX* members (Table S1). Following the established classification framework for *BBX* genes in *C. ensifolium*, we grouped these genes into four distinct clades, with Group II showing the highest number of members. This suggests a possible expansion and functional diversification within this clade specific to *C. ensifolium*.

Gene duplication events are pivotal in the evolutionary dynamics of plant genomes, with both small-scale duplications (e.g., tandem gene duplication) and large-scale duplications (e.g., whole-genome and segmental duplications) driving these processes [56]. The relatively lower number of *BBX* genes in *C. ensifolium* compared to other species may be attributable to two whole genome duplication events within the orchid lineage [57]. Our findings suggest that both small-scale duplications and large-scale duplications have significantly influenced the evolution and diversification of the *BBX* gene family in *C. ensifolium*, reflecting evolutionary patterns observed in other plant species.

*BBX* genes play diverse functional roles in various biological processes within plants [34]. Mounting evidence suggests that the biological functions of *BBX* genes are strongly linked to their particular expression patterns. Gene expression is controlled by cis-regulatory elements located in promoter regions. These elements function as molecular switches that allow transcripts to be involved in intricate regulatory networks [58]. Our examination of cis-acting elements in the *BBX* promoters indicates a significant concentration of photoresponsive elements in the promoter regions of *CeBBX* genes. Additionally, plant hormone-responsive cis-elements were widely distributed, indicating that *BBX* gene expression is modulated not only by light but also by internal hormone levels, enabling the plant to respond to external environmental cues and developmental processes.

Additionally, differential expression analysis under blue light treatment underscored the functional diversity of *CeBBX* genes. *CeBBX8* and *CeBBX14* were significantly upregulated under blue light, while *CeBBX3* and *CeBBX15* were significantly downregulated, suggesting their involvement in the blue-mediated pathway and their potential roles in stress response and light morphogenesis. Interestingly, the upregulation of *CeBBX14* in response to blue light was not observed in the fluorescence quantitative experiments, possibly due to circadian rhythm influences, which aligns with findings from *FvCO* studies [35]. Furthermore, *CeBBX14* is a homologous protein to *RoCO*, which regulates the flowering time in roses, thereby emphasizing the evolutionary conservation and functional significance of *BBX* genes across species [59].

## 5. Conclusions

In this study, we discovered and examined the *BBX* gene family within the genome of *C. ensifolium*. Detailed investigations were conducted to elucidate the gene structures, conserved patterns, regulatory elements, chromosomal placements, and similarities with genes from different species. Our results highlight, under artificial control, the strong response of *BBX* genes to blue light during meristem induction in *C. ensifolium*. This study establishes a foundational framework for understanding the meristem induction of *C. ensifolium* by artificial light control, contributing to a broader comprehension of gene functions in orchids. These investigations will provide deeper insights into their contributions to the phenotypic traits of *C. ensifolium* and related species, thereby enhancing our ability to utilize these genes for horticultural and agricultural advancements.

## Data Availability

All data generated or analyzed during this study are included in this published article (Supplementary Materials) and available from the corresponding author upon reasonable request.

## Supplementary Materials

The following supporting information can be downloaded at: https://www.mdpi.com/article/10.3390/plants13172375/s1, Table S1 *BBX* gene family identified in *Cymbidium ensifolium*; Table S2 Prediction of protein secondary structure of *BBX* gene.
