# Supplementary material for: BBX Genes of Cymbidium ensifolium Exhibited Intense Response to Blue Light in Meristem Induction through Artificial Control
Source: Plants (Basel). 2024 Aug 26;13(17):2375. doi: 10.3390/plants13172375 (PMC11396916; doi:10.3390/plants13172375)
Supplement: Supplementary file 1 [file plants-13-02375-s001.zip › plants-3137845-supplementary.pdf]

Supplement Table S1 *BBX* gene family identified in *Cymbidium ensifolium*

Sequence ID	Gene ID	Number of Amino Acid	Molecular Weight	Theoretic-al pI	Instability Index	Aliphatic Index	Grand Average of Hydropathi-city	Subcellular Localization
*CeBBX*1	JL001290	250	28135.7	4.87	53.91	69	-0.304	Nucleus
*CeBBX*2	JL021791	325	34306.61	5.93	45.34	68.62	-0.199	Nucleus
*CeBBX*3	JL004927	428	47499.04	6.85	49.38	68.64	-0.569	Nucleus
*CeBBX*4	JL008902	243	26434.1	4.89	47.96	86.79	-0.11	Nucleus
*CeBBX*5	JL010714	241	26486.85	5.4	40.06	64.52	-0.389	Nucleus
*CeBBX*6	JL008161	443	47951.09	6.14	52.79	65.91	-0.509	Nucleus
*CeBBX*7	JL011233	416	45132.05	5.38	51.77	61.9	-0.446	Nucleus
*CeBBX*8	JL008605	276	30860.63	5.08	59.93	70.69	-0.503	Nucleus
*CeBBX*9	JL012712	285	30862.02	6.11	48.75	81.19	-0.159	Nucleus
*CeBBX*10	JL010233	189	20281.83	7.11	51.83	71.32	-0.254	Nucleus
*CeBBX*11	JL010243	259	28670.47	6.2	59.7	73.86	-0.436	Nucleus
*CeBBX*12	JL010830	284	30820.85	5.3	47.54	68.06	-0.237	Nucleus
*CeBBX*13	JL012311	363	41023.43	8.01	63	59.42	-0.755	Nucleus
*CeBBX*14	JL012445	330	35122.54	6.98	50.87	66.67	-0.173	Nucleus
*CeBBX*15	JL023300	587	64770.75	7.22	48.47	67.87	-0.508	Nucleus
*CeBBX*16	JL013155	337	37473.09	5.14	54.6	74.39	-0.425	Nucleus
*CeBBX*17	JL013922	384	42181.46	5.53	62.16	64.58	-0.548	Nucleus
*CeBBX*18	JL018006	295	32285.95	5.17	58.93	60.58	-0.486	Nucleus
*CeBBX*19	JL021624	242	27546.45	6.7	46.62	70.04	-0.511	Nucleus

Supplement Table S2 Prediction of protein secondary structure of *BBX* gene

	Alpha helix	Extended strand	Beta turn	Random coil
CeBBX1	19.60%	17.60%	6.40%	56.40%
CeBBX2	35.08%	11.38%	3.69%	49.85%
CeBBX3	23.13%	5.84%	0.00%	71.03%
CeBBX4	27.57%	12.35%	4.53%	55.56%
CeBBX5	26.97%	13.69%	2.90%	56.43%
CeBBX6	20.99%	6.77%	0.00%	72.23%
CeBBX7	24.04%	10.34%	3.37%	62.26%
CeBBX8	26.40%	13.79%	5.37%	54.44%
CeBBX9	29.82%	14.39%	5.96%	49.82%
CeBBX10	21.16%	17.46%	1.59%	58.79%
CeBBX11	23.02%	11.96%	2.93%	62.08%
CeBBX12	19.37%	13.38%	3.17%	64.08%
CeBBX13	38.57%	7.71%	3.31%	50.41%
CeBBX14	31.52%	11.82%	1.52%	55.15%
CeBBX15	29.13%	12.10%	3.75%	55.03%
CeBBX16	19.29%	11.87%	1.48%	67.36%
CeBBX17	39.06%	11.46%	4.43%	45.05%
CeBBX18	20.85%	16.22%	3.09%	59.85%
CeBBX19	18.60%	14.88%	2.89%	63.64%
